# Multidisciplinary evaluation of the remineralization potential of three fluoride-based toothpastes on natural white spot lesions

**DOI:** 10.1007/s00784-023-05334-2

**Published:** 2023-10-19

**Authors:** Giulia Orilisi, Flavia Vitiello, Valentina Notarstefano, Michele Furlani, Nicole Riberti, Riccardo Monterubbianesi, Tiziano Bellezze, Guglielmo Campus, Florence Carrouel, Giovanna Orsini, Angelo Putignano

**Affiliations:** 1 Department of Clinical Sciences and Stomatology (DISCO), Università Politecnica Delle Marche, 60126 Ancona, Italy; 2 Health, Systemic, Process (P2S), Research Unit UR 4129, University Claude Bernard Lyon 1, University of Lyon, 69008 Lyon, France; 3 Department of Life and Environmental Sciences, Università Politecnica Delle Marche (DISVA), 60131 Ancona, Italy; 4 Department of Neurosciences Imaging and Clinical Sciences (DNISC), University of Chieti-Pescara, 66100 Chieti, Italy; 5 Department of Materials, Environmental Sciences and Urban Planning (SIMAU), Università Politecnica Delle Marche, 60131 Ancona, Italy; 6 Department of Surgery, Microsurgery and Medicine Sciences, School of Dentistry, University of Sassari, 07100 Sassari, Italy; 7 Department of Restorative, Preventive and Pediatric Dentistry, University of Bern, CH-3010 Bern, Switzerland; 8 National Institute of Health and Science of Aging (INRCA), 60124 Ancona, Italy

**Keywords:** Remineralizing agents, Toothpaste, Fluoride, Hydroxyapatite, White spot lesions, Multidisciplinary analysis

## Abstract

**Objectives:**

This in vitro study aimed assessing the remineralization potential of three commercial fluoride-based toothpastes in permanent teeth with natural white spot lesions (WSLs). A multidisciplinary approach based on Raman microspectroscopy (RMS), Scanning electron microscopy (SEM), Energy-dispersive x-ray spectroscopy (EDS), and Vickers microhardness (VMH) was exploited.

**Methods:**

*N* = 12 human molars with natural WSLs in the proximal-vestibular zone were selected and divided into 4 groups (*n* = 3) according to the different treatments: HAF (hydroxyapatite with fluoride ions); SMF (sodium monofluorophosphate with arginine); SF (sodium fluoride with enzymes), and CTRL (untreated group). All toothpastes tested contained 1450 ppm of fluoride. Teeth samples were submitted to the following protocol: a 7-day pH cycling treatment, with two daily exposures (2 min each time) to the commercial toothpastes described above. The surface micromorphology (SEM), the chemical/elemental composition (RMS and EDS), and the Vickers microhardness (VMH) were evaluated. Statistical analysis was performed.

**Results:**

A remarkable remineralization of WSLs in SEM images was observed in all treated groups compared to CTRL. In particular, HAF and SF displayed higher values of VMH, phosphates amount (I_960_), crystallinity (FWHM_960_), and lower ones of C/P (I_1070_/I_960_) with respect to CTRL. Intermediate values were found in SMF, higher than CTRL but lower with respect to HAF and SF. As regards the Ca/P ratio, statistically significant differences (*p* < 0.05) were found between SF and the other groups.

**Conclusions:**

All the tested dentifrices have shown to remineralize the WSLs. SF and HAF have comparable capability in hardness recovery and crystallinity; however, SF shows the best remineralizing potential according to both micromorphological and chemical analyses.

Clinical relevance

The daily use of toothpastes containing hydroxyapatite partially replaced with fluoride, sodium monofluorophosphate with arginine and sodium fluoride toothpaste associated with enzymes represents a preventive, therapeutic, effective, and non-invasive tool for remineralize WSLs.

## Introduction

Tooth caries represents a growing problem in modern dentistry, and, its clinical management should be focused not only on the treatment but also on its early detection and prevention. Luckily, caries formation has a slow progression, and therefore the reversal mechanism can be activated if the lesion is detected early and managed adequately. The demineralization of hard dental tissues is a very complex process due to the action of acid-producing bacteria present in the oral micro-environment, which causes the accumulation of numerous episodes of demineralization and remineralization [1–3]. Indeed, the outermost zone of tooth enamel comes into close contact with saliva and plaque fluid. As a result, hydroxyapatite (HA) crystals existing on the enamel’s surface maintain a dynamic balance with these nearby aqueous phases [4]. The acidic attacks from oral biofilms cause the dissolution of HA into Ca^2+^ and PO_4_^3−^ ions [5] and clinically lead to the first stage of the carious disease, named white spot lesion (WSL), which is characterized by enamel demineralization without cavitation [1].

Saliva is a determining biological factor in dental caries which plays many roles including neutralizing the acid, providing mineral ions to assist remineralization, and forming the protective acquired enamel pellicle [6, 7]. The process of de- and remineralization is influenced by the saturation level of oral fluids (saliva and plaque) [8]. Under suitable changes in conditions, remineralization can become the prevailing process, thereby facilitating to lesion repair [9].

Remineralization of WSLs is thought to be a tool that could close the gap between prevention and clinical procedures, and, in recent years, many efforts have been made to improve already existing clinical protocols and introduce new ones [1, 10, 11]. To this purpose, different treatments have been developed based on specific active agents, including fluoride, nano-HA, bioactive glass, casein phosphopeptide-amorphous calcium phosphate, and infiltrative resins [12–15].

Several techniques are available to explore the demineralization and remineralization processes. Besides Vickers microhardness (VMH) analysis, advanced high-resolution 2D and 3D analytical techniques are available, including Scanning electron microscopy (SEM), Energy-dispersive x-ray spectroscopy (EDS), Computed x-ray microtomography (µ-CT), and Raman microspectroscopy (RMS) [16–19]. The coupling of all these innovative techniques lets us obtain a complete overview of hard dental tissues, providing morphological, volumetric, elemental, and chemical information on teeth mineralized tissues [20–24]. µ-CT represents a powerful and non-destructive tool to study the demineralization and remineralization of teeth and to evaluate the mineral density; furthermore, this high-resolution technique provides volumetric data without the need to slice the sample [20, 21]. RMS is a non-destructive and label-free vibrational technique that has been employed, in the last years, by an increasing number of researchers, to study mineralized tissues, such as bones and teeth, since it is sensitive both to the mineral and the organic components [22–24].

Based on the increasing request for more performant remineralization agents, in this in vitro study, the remineralization potential of three different commercial fluoride-toothpastes on permanent human molars with natural WSLs has been investigated by exploiting a multidisciplinary approach based on RMS, µ-CT, SEM, EDS, and VMH. The proposed null hypothesis (H0) was that the tested materials did not show differences in the remineralizing potential of WSLs.

## Materials and methods

### Sample collection

Permanent human molars (*n* = 12) were collected at the Department of Clinical Sciences and Stomatology (DISCO) of Università Politecnica delle Marche (Ancona, Italy). Teeth were surgically extracted for therapeutic purposes. In accordance with the guidelines of the Local Ethical Committee and the WMA-Declaration of Helsinki (2018) [25], informed consent was obtained from all patients, ensuring they were fully aware that their hard dental tissues would be used for research purposes.

**Fig. 1:**
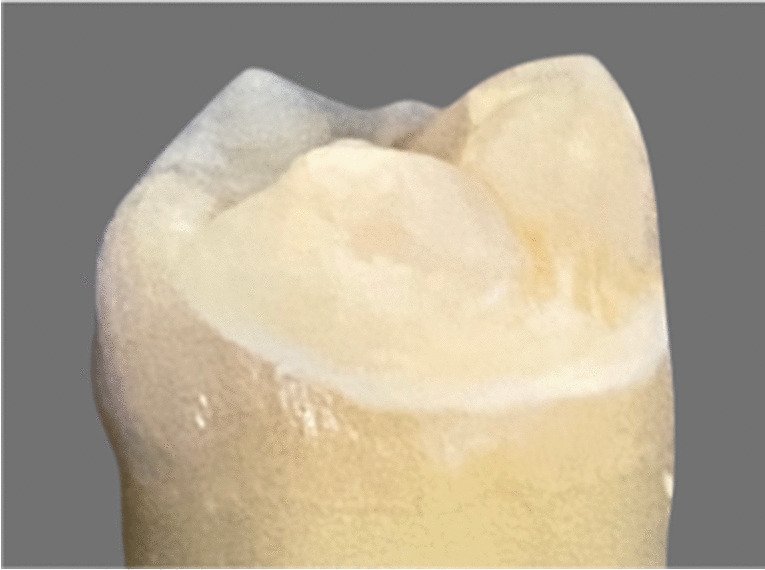
Representative image of a natural WSL used in the study

Following the surgical extraction, the specimens underwent a 2-min cleaning in an ultrasonic bath with distilled water, in order to remove blood and biological remains. All tooth samples were visually inspected by two experienced clinicians (GiuO and FV), who selected only those in which the presence of areas white in color with no surface damage attributable to natural WSLs in the proximal-vestibular zone was detected (Fig. 1). Samples were stored in artificial saliva (Biotene Oralbalance Gel, GSK, England) before the start of the treatments.

After the collection of teeth, samples were divided into 4 groups (*n* = 3 teeth per group) and treated for 7 days with different commercial fluoride-based toothpastes, according to the assigned group:

- Group HAF: hydroxyapatite partially replaced with fluoride, 1450 ppm F^−^;
- Group SMF: sodium monofluorophosphate with arginine, 1450 ppm F^−^;
- Group SF: sodium fluoride with enzymes, 1450 ppm F^−^;
- Group CTRL: WSL not treated.

The composition of the toothpastes used in this study is reported in Table 1.

**Table 1:** Remineralizing agents used in the study

Group	Product	Ingredients	Active agents	Manufacturer
HAF	Curasept Biosmalto	Purified water, glycerin, hydrated silica, MgSrcarbonate hydroxyapatite conjugated with chitosan, cellulose gum, xylitol, cocamidopropyl betaine, aroma, sodium, xhantam gum, potassium acesulfame, ethylhexylglycerin, phenoxyethanol, sodium benzoate, citric acid	1450 ppm F^−^ (as hydroxyapatite partially replaced with fluoride)	Curasept® S.p.a, Italy
SMF	Elmex Caries Professional	Calcium carbonate, water, glycerin, sodium lauryl sulfate, arginine, aroma, cellulose gum, sodium bicarbonate, tetrasodium pyrophosphate, benzyl alcohol, sodium saccharin, sodium hydroxide, CI 77891	1450 ppm F^−^ (as sodium monofluorophosphate)	Elmex®, Colgate-Palmolive Manufacturing, Swidnica, Poland
SF	Curaprox Enzycal 1450	Water, sorbitol, hydrated silica, glycerin, steareth-20, titanium dioxide, aroma, sodium hydrogenphosphate, carrageenan, sodium chloride, citric acid, sodium benzoate, sodium saccharin, potassium thiocyanate, enzymes (glucose oxidase, amyloglucosidase, lactoperoxidase)	1450 ppm F^−^ (as sodium fluoride)	Curaden® AG, Kriens, Switzerland

### Sample preparation

**Fig. 2:**
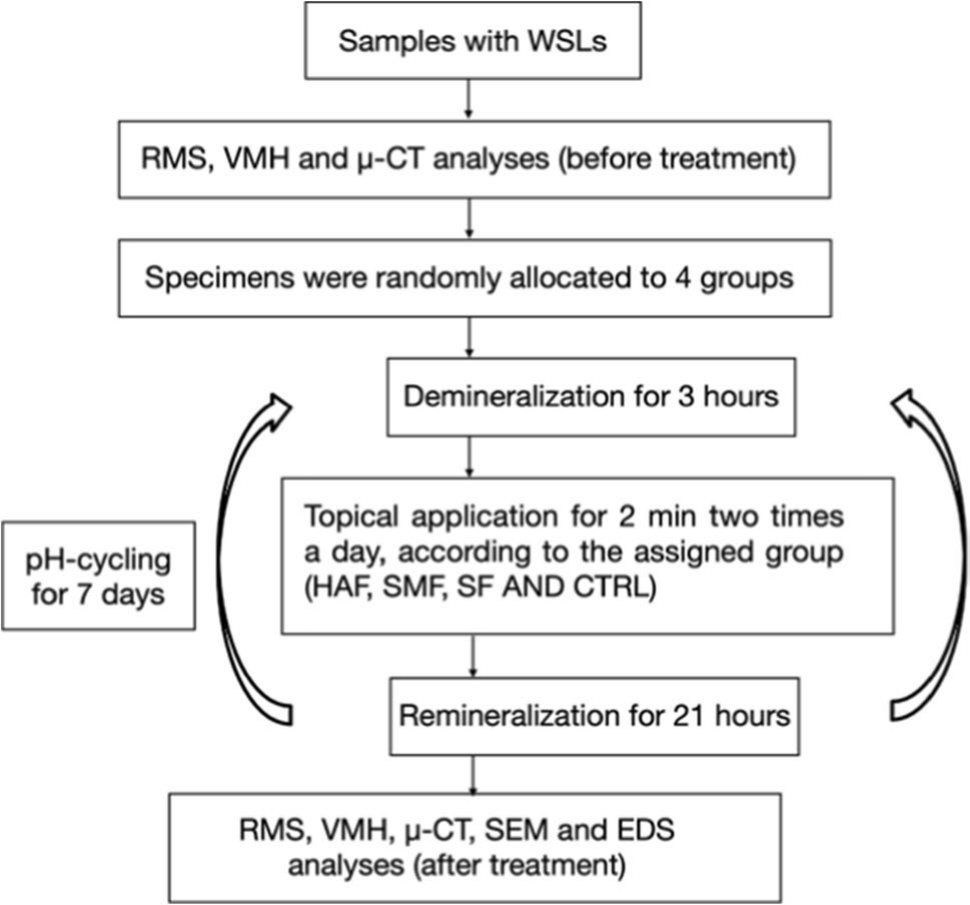
Flowchart of the experimental design of the study

Samples were embedded in epoxy resin circular molds with 16 mm diameter and 16 mm deep (Technovit, Kulzer Technik, Wehrheim, Germany), with the enamel affected area exposed for the analyses [26]. For each sample, the following measurements were recorded at the baseline and after 7 days of treatment: RMS, VMH, and µ-CT, while SEM and EDS analyses were performed only after 7 days of treatment since these techniques could damage the samples. A schematic flow chart of the study’s procedures is presented in Fig. 2.

### pH-cycling and treatment with toothpastes

Samples were subjected to a pH-cycling protocol of alternative demineralization and remineralization solutions for 7 consecutive days in order to simulate pH variations that occur in the oral microenvironment [27, 28]. In a 24 h-cycle, samples were immersed individually in remineralization solution (1.5 mML^−1^ calcium, 0.9 mML^−1^ phosphate, 150 mML^−1^ potassium chloride in 0.02 mML^−1^ cacodylic buffer, 0.02 μgF/mL, and 1 mL/mm^2^) (pH = 7) for 21 h and immersed in demineralization solution (2.0 mML^−1^ calcium and phosphate in 75 mML^−1^ acetate buffer, 0.03 μgF/mL and 3 mL/mm^2^) (pH = 4.3) for 3 h. Samples received twice a day 2 min treatments, according to the assigned group, with the toothpaste slurry with a soft toothbrush (Curaprox CS 5460, Curaden® AG, Kriens, Switzerland). The toothpaste slurry was prepared by mixing for 30 s in a beaker with a cylinder-shape magnetic stirring bar a peanut-sized toothpaste (equivalent to the volume of a standardized toothpaste lid) with the artificial saliva (Biotene Oralbalance Gel, GSK, England) (three times volume of the toothpaste) [8]. After each step, the samples were rinsed with deionized water for 5 s. The solutions were replenished every 24 h.

### VMH measurements

All samples’ surface microhardness was measured using the VMH tester Remet HX-1000 (Remet S.A.S., Casalecchio di Reno, Italy). To standardize the measurements and reduce the error due to the curved surface of the tooth, each sample was embedded in a resin block with WSL area exposed, as previously described. On each selected area, three indentations were performed using a pyramid-shaped diamond indenter, loaded with 100 g for 15 s; the distance between one indentation and another was approximately 100 µm. After removing the load, the values of the indentation diagonals were evaluated by using a microscope and the Proximo ver. 9 software, which automatically determines the Vickers microhardness number (HV number, expressed as Kg/mm^2^). Data were reported as mean ± standard deviation. The hardness was assessed both before (HVpre) and after (HVpost) the treatment in the pH-cycling and remineralization process. The percentage of remineralization potential (% RP) was calculated according to the following equation: %RP = [(HVpost–HVpre)/HVpre] × 100 [26].

### RMS measurements and data analysis

A Horiba Jobin–Yvon XploRA Nano Raman microspectrometer, equipped with a 785-nm diode laser, was used as a source. All RMS measurements were acquired by using a 5 × objective (Olympus, Tokyo, Japan). Before acquiring the spectra, the spectrometer was calibrated to the 520.7 cm^−1^ line of silicon. A 600 lines per mm grating was chosen. A 200 μm confocal pinhole was used for all measurements. The spectra were dispersed onto a 16-bit dynamic range Peltier-cooled CCD detector. Raman maps were acquired on rectangular areas (241.5 µm × 167 µm), at the interface between the WSL and the sound enamel. A step size of ~ 15 µm was adopted for a total number of 192 spectra. From each Raman map, the average spectrum of the WSL region was extracted and submitted to preprocessing procedures, including baseline correction (2 iterations, polynomial method), smoothing (5 points), and vector normalization (OPUS 7.5 software). The area (A), intensity (I), and full width at half maximum (FWHM) of the band centered at 960 cm^−1^ (spectral range 929–976 cm^−1^), assigned to the stretching of PO_4_^3−^ groups of HA (A_960_, I_960_, and FWHM_960_) [29, 30], and the intensity (I) of the band centered at 1070 cm^−1^ (spectral range 1051–1088 cm^−1^), assigned to the stretching of CO_3_^2−^ groups (I_1070_) [22, 29, 31] were calculated (Labspec 6 software, Horiba Scientific) [22, 29].

### µ-CT acquisition and reconstruction

µ-CT analysis was performed on SF and CTRL samples, to deepen the results obtained from RMS and VMH analyses. A µ-CT system Bruker SkyScan 1174 (SkyScan-Bruker, Antwerp, Belgium) was used. Projection’s settings were as follows: acceleration voltage 50 kV; beam current 800 μA; aluminum filter of 1 mm; pixel size 11.5 μm and rotation 180° in 0.3° step with an exposure time of 10 s per projection. The average scan time was 2 h. A total number of 830 reconstructed sections were obtained for each sample, providing axial information covering an approximate tooth thickness of 12 mm. NRecon software (Version 1.6.10.2, Bruker, Billerica, MA, USA) was used to convert the tooth projections into cross-sectional slices, with the following correction settings: Ring artifacts (7.0); smoothing (6.0); beam hardening (40%), and proper misalignment compensation.

The mineral density (MD) was quantified, using a fitting curve, by the equation MD = 0.00972 ×  + 0.98654. This curve was properly obtained (unpublished data). Gray values, ranged from 0 to 255, were evaluated using the histogram function in square regions inside the WSL of SF and CTRL in five different slices.

### SEM and EDS evaluation

SEM and EDS measurements were performed by means of a Zeiss Supra 40 field-emission electron microscope (Zeiss, Oberkochen, Germany). Before imaging, all specimens were meticulously cleaned, dehydrated, and positioned in a sample holder. Furthermore, a vacuum deposition process was performed to apply a gold film on the dental surface. SEM micrographs were captured at magnifications of 2000 × and 7000 × , with the SEM operating at 20 kV and at a 7 mm working distance. The acquired micrographs were employed for evaluating the micromorphology and elemental composition of the WSLs before and after treatment. The elemental surface characterization was conducted using EDS with the EDAX Element Microanalysis (AMETEK Gmbh, EDAX Business Unit, Weiterstadt, Germany) [16]. Three points per sample were randomly selected, and the mean values were calculated. EDS analyses were performed under the following operating parameters: a working distance of 15 mm, an acceleration voltage of 20 kV, and a magnification of 1000 × . The degree of mineralization was evaluated by quantifying the amounts of phosphorus (P) and calcium (Ca) and calculating their ratio (Ca/P). The results are presented as the mean value and standard deviation.

### Statistical analysis

Sample size was determined based on preliminary hardness results before and after treatment via the ANOVA, using the software G*Power 3.1.9 (Franz Faul, Kiel University, Kiel, Germany) at a power of 0.8 and a statistical significance of 0.05 [32]. The effect size and the correlation among repeated measures were 0.5 and 0.7, respectively. This calculation indicated that three samples per group were required.

After homogeneity and normality evaluations, one-way analysis of variance (ANOVA) and the post hoc *t*-test and Tukey’s HSD test were used for the statistical comparison (*α* = 0.05). Statistical analyses were performed with the software package Prism6 (Graphpad Software, Inc. USA). All data were presented as mean ± standard deviation (SD). Statistical significance was set at *p* < 0.05.

## Results

### Surface microhardness

**Fig. 3:**
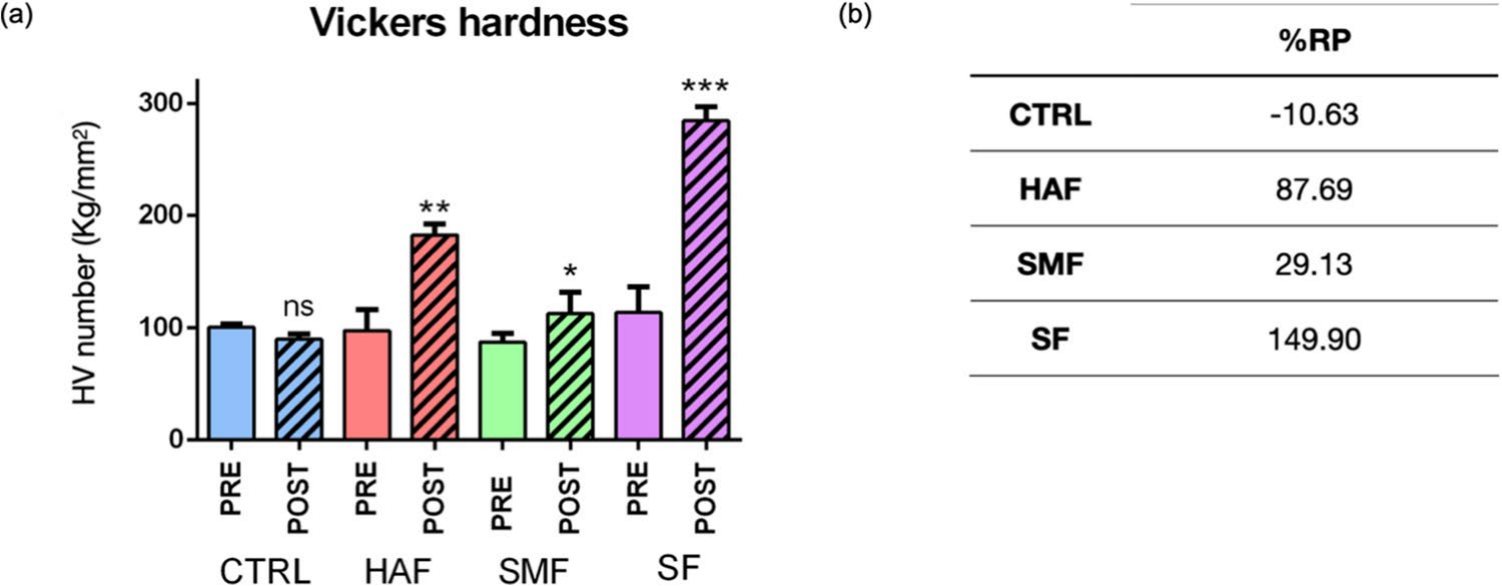
Microhardness values (**a**) and percentage of remineralization potential (% RP) (**b**), calculated for all the experimental groups. Data are presented as mean ± standard deviation. Statistical significance among HV-PRE and POST values for each group was evaluated using Student’s *t*-test. Statistical significance was set at *p* < 0.05 (ns, not significant; *p < 0.05, **p < 0.01, and ***p < 0.001)

The microhardness values are reported in Fig. 3a. Before the remineralizing treatments all groups evidenced similar microhardness values. Comparing HVpost values with HVpre ones, in groups HAF (*p* = 0.0022), SMF (*p* = 0.0257), and SF (*p* = 0.0003) HVpost values increased with respect to HVpre one. On the contrary, CTRL highlighted a lower reduction not statistically significant (*p* = 0.0651). After 7 days of treatment, HAF and SF showed statistically significant higher values (182.88 ± 9.63 and 248.44 ± 12.55, respectively) than SMF (112.78 ± 19.18) (*p* < 0.05). Moreover, to better elucidate the differences in remineralization potential between experimental groups, %RP was calculated. The following decreasing order was found in SF, HAF, SMF, and CTRL, as shown in the table in Fig. 3b. In particular, between the treated groups, %RP was lowest in SMF and highest in SF, showing the major capability of remineralization.

### Raman microspectroscopy

**Fig. 4:**
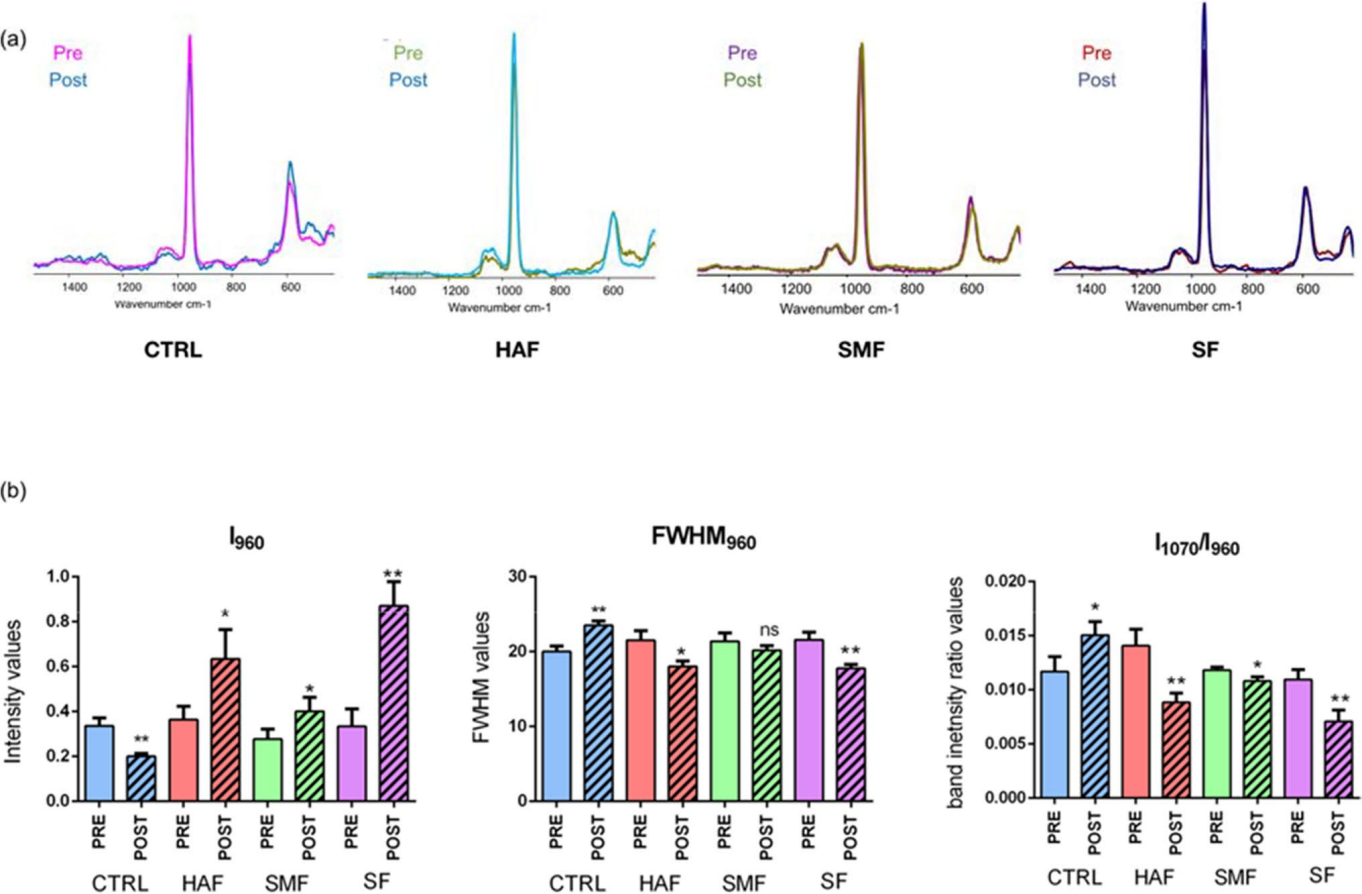
**a** Raman spectra of WSLs before and after the remineralizing treatments of the following groups: CTRL, HAF, SMF, and SF. **b** Statistical analysis of the following spectral parameters calculated on CTRL, HAF, SMF, and SF white spot lesions before (pre) and after (post) remineralization treatments: I_960_ (phosphate groups), FWHM_960_ (inversely proportional to Crystallinity), and I_1070_/I_960_ (carbonates/phosphates). Data are presented as mean ± standard deviation. Statistical significance among groups (pre and post) was evaluated using Student’s *t*-test. Statistical significance was set at *p* < 0.05 (*p < 0.05; and **p < 0.01)

All samples were also submitted to RMS analysis, and the Raman spectra of all samples before and after the remineralizing treatments are displayed in Fig. 4a. Noteworthy, a decrease of the peak of the peak at 960 cm^−1^, associated with the stretching vibration of PO_4_^3−^ groups in HA, was highlighted in CTRL after the pH-cycling; in SMF, the peak remained unchanged after treatment while an increase was detected in HAF and SF. To better highlight changes in the chemical composition of the WSL lesions after the remineralization treatments, specific spectral parameters were calculated and statistically analyzed (Fig. 4b). CTRL samples were characterized by a statistically significant lower amount of PO_4_^3−^ groups in HA (I_960_), because of the pH cycling protocol (*p* = 0.0034). Conversely, in all treated samples, which were submitted both to pH cycling and remineralizing treatments, higher values were observed, mainly in HAF (*p* = 0.0302) and SF (*p* = 0.0022). Crystallinity is inversely proportional to FWHM_960_, which means that a narrow bandwidth denotes high mineral crystallinity, and vice versa. Also, in this case, after the pH cycling, CTRL samples displayed higher FWHM_960_ values (*p* = 0.0031) and hence a lower crystallinity. As regards treated samples, HAF and SF presented lower FWHM_960_ post values with respect to the pre ones and hence a higher crystallinity (respectively, *p* = 0.0138 and *p* = 0.0043), while no statistically significant difference was found in SMF before and after treatment (*p* = 0.1818). Finally, a higher amount of carbonate to phosphate groups (band intensity ratio I_1070_/I_960_; C/P) was detected in CTRL samples after the pH cycling (*p* = 0.0355). In all treated samples, lower values of I_1070_/I_960_ were found after the treatment, mainly in HAF and SF (respectively, *p* = 0.0070 and *p* = 0.0082). Thus, a significant remineralization of WSLs was observed mainly in HAF- and SF-treated groups compared to CTRL.

### µ-CT and mineral density

**Fig. 5:**
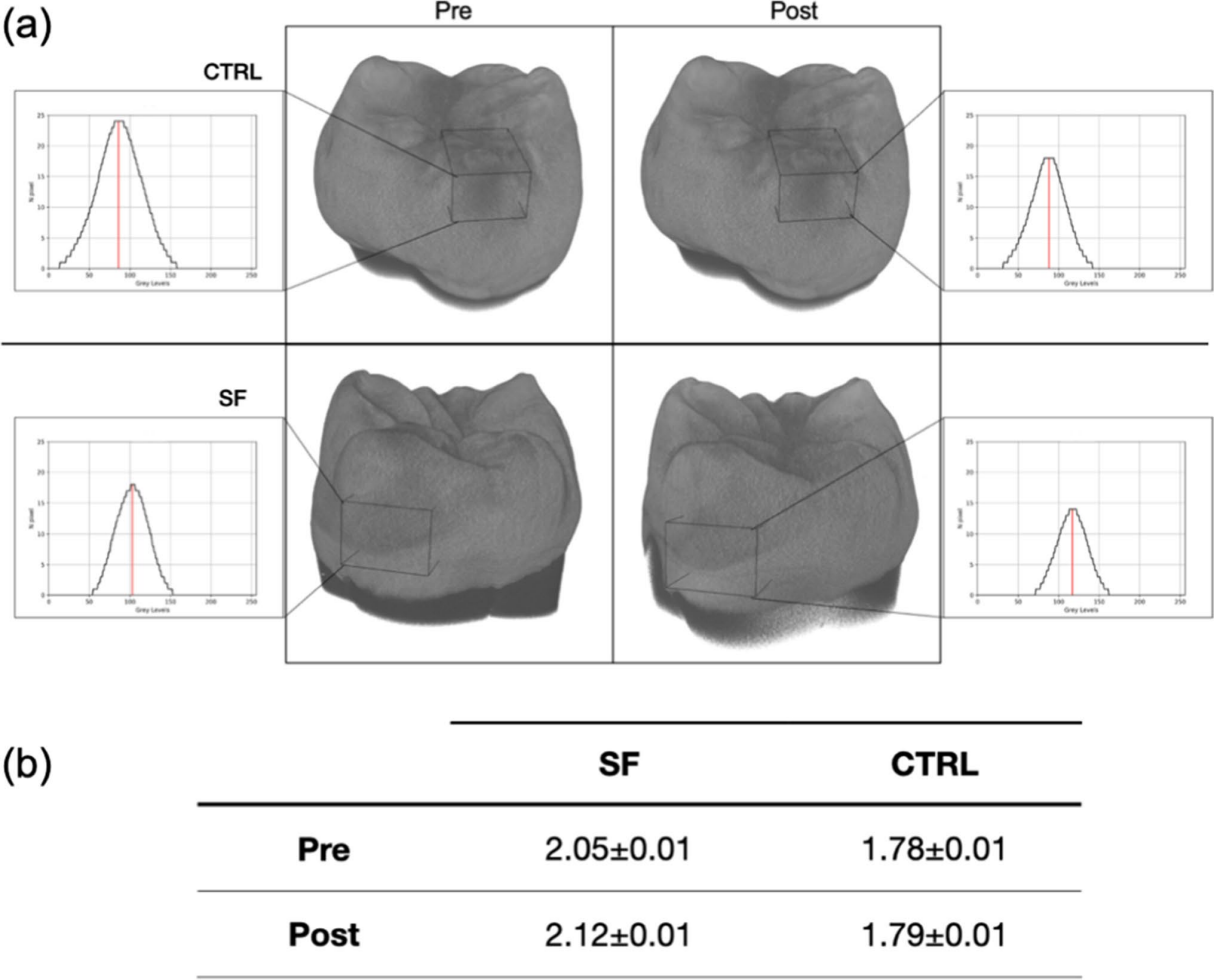
**a** µ-CT 3D reconstructions of a representative sample of both SF and CTRL groups before (pre) and after (post) 7 days. Samples were oriented to better visualize the WSL region (darker regions inside the black boxes). **b** Mineral density (g/cm^3^) values of WSLs were reported as mean ± standard deviation for each group, before and after the treatment, for comparison

Based on the results collected by RMS measurements, µ-CT analysis was performed on a representative sample of SF, which showed from the previous analyses the best performance in terms of remineralization, and CTRL group, to better explore the remineralization effects in terms of MD values (Fig. 5). The 3D reconstructions highlighted large variations in shape and size (Fig. 5a) of the WSL in SF after treatment, with also an increase of MD values (Fig. 5b) obtained from the fitting curve applied to the gray levels measured in the WSL region (delimited by black boxes in Fig. 5a) before and after the treatment. No meaningful differences are appreciable in the CTRL group, neither for size and shape (Fig. 5a) nor for the MD values obtained from the fitting curve as described above (Fig. 5b).

### SEM observations and EDS analysis

**Fig. 6:**
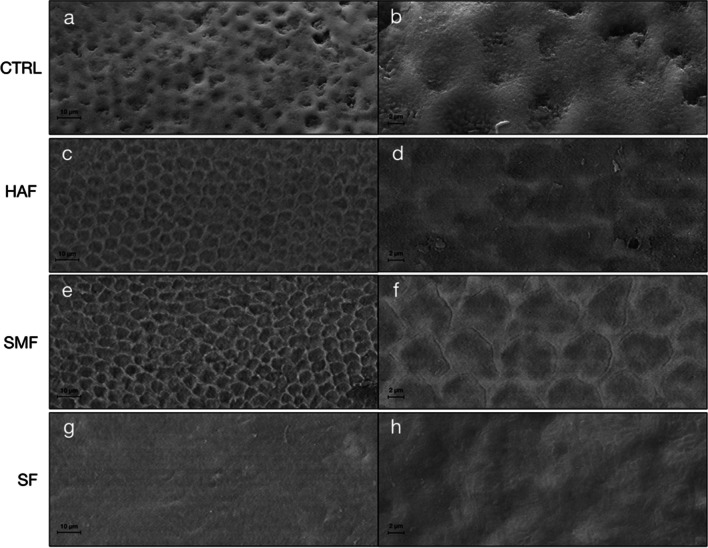
Scanning electron micrographs of a representative sample of each group at 2000 × and 7000 × magnifications, after 7 days of treatment. SEM micrographs of CTRL group (**a**, **b**) highlighted an enamel demineralization morphology with selective dissolution of the apatite crystals inside the prisms. In HAF (**c**, **d**) and SMF (**e**, **f**) groups, a partial remineralization with partial restoration of the interprismatic areas was observed; while, in SF group (**g**, **h**) a complete remineralization with complete restoration of the interprismatic area was highlighted. HAF group, hydroxyapatite partially substituted with fluoride; SMF group, sodium monofluorophosphate with arginine; SF group, sodium fluoride; CTRL, untreated group

SEM micrographs were performed on all experimental groups after treatment, at 2000 × and 7000 × magnifications for each treated group, as shown in Fig. 6. CTRL showed an irregular pattern of slits and roughness surface with high evidence of porosities (Fig. 6a, b), due to the mineral loss in rod–interrod junction and within the rod structure. These observations indicated no remineralization occurred after the pH-cycling process. The SEM micrograph of HAF showed, especially at high magnification, a quite smooth surface, suggesting the formation of a new apatite layer and remineralization of the lesion (Fig. 6c, d). On the contrary, the SEM micrograph of SMF exhibited a slight roughness surface in some areas, more visible at low magnification, which indicated that remineralization took place but not completely (Fig. 6e, f). Finally, SF micrographs evidenced, both at 2000 × and 7000 × magnification, the most homogenous and regular surface, compared to HAF and SMF. In particular, with respect to the micrograph of CTRL (Fig. 6a, b), a reconstitution of the typical prismatic enamel structure occurred (Fig. 6g, h).

**Fig. 7:**
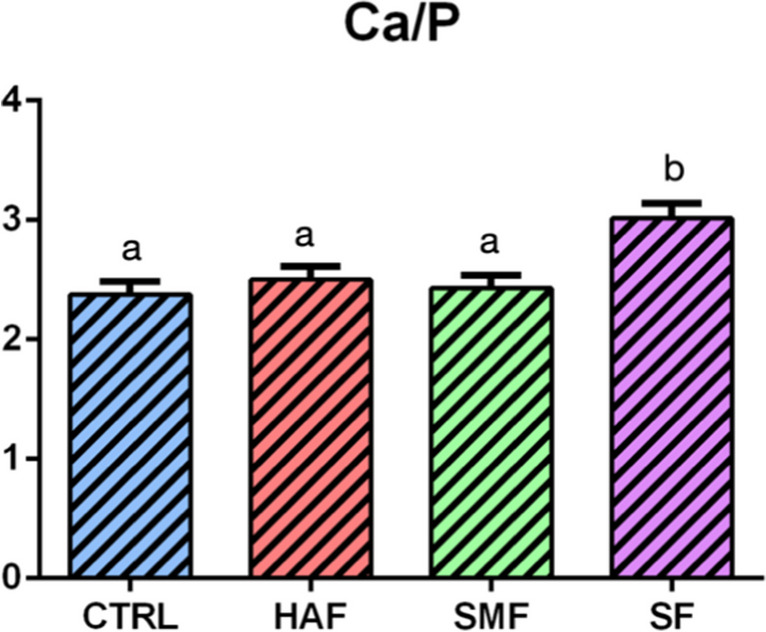
Mean values and standard deviation of Ca/P ratio, according to the treatment groups. Statistical significance among groups was evaluated using Tukey’s HSD test. Lower-case letters over histograms indicate statistically significance differences between the groups (*p* < 0.0001). HAF group, hydroxyapatite partially substituted with fluoride; SMF group, sodium monofluorophosphate with arginine; SF group, sodium fluoride; CTRL, untreated group

EDS analysis, performed after treatment, detected different amounts of percentages in weight (%wt) of Ca and P between the tested groups, confirmed by the Ca/P ratio (Fig. 7). In particular, CTRL displayed the lowest Ca/P mean ratio (2.37 ± 0.11), while SF showed the highest one (3.01 ± 0.12). HAF and SMF highlighted similar values, respectively 2.50 ± 0.11 and 2.43 ± 0.11. However, only SF displayed statistically significant differences wih respect to the other groups (*p* < 0.0001).

## Discussion

Nowadays, in modern restorative dentistry, the remineralization can be considered a preventive strategy and a minimally invasive therapeutic approach for initial carious enamel lesions [15]. Many efforts were made to provide efficient therapies in terms of remineralization and aesthetic appearance of WSL, such as fluorides, phosphopeptide compounds, xylitol, infiltrative resins, microabrasion, and/or bleaching and preparation and restoration [33]. However, although various therapies have been proposed for WSLs, no specific intervention has been selected as a perfect solution for this commonplace trouble [4, 34]. Consequently, WSLs continue to present a significant challenge for both clinicians and patients. Given the high prevalence of WSLs and erosion in individuals, in particular adolescents, there is a need to deepen the knowledge on preventive and non-invasive treatments [35]. Thus, the objective of this study was to compare the effectiveness of remineralization of three fluoride-based toothpastes.

In order to reproduce the daily routine, the brushing procedures, used in this study, were accomplished by brushing the specimens with toothpaste/remineralizing solution slurry for 120 s, considered as total contact time of the slurry, twice daily [8]. Rationale for using this procedure was to be as faithful as possible to clinical practice, in which toothpaste is diluted in the salivary secretion [36]. Thus, one part of toothpaste was dissolved in three parts (1:3) of artificial saliva to obtain a homogeneous slurry. Moreover, pH values represent a key factor during the demineralization and remineralization process. Indeed, previous studies reported that only pH values higher than 5.5 have been assumed to promote lesion arrest and to facilitate remineralization [37]. In this light, samples were subjected to pH-cycling, in order to mimic the dynamics of pH fluctuations which occurred physiologically in the oral cavity [38].

The results of the present study demonstrated that all the three tasted fluoride-toothpastes favored remineralization on natural WSL after 7 days of treatment, in comparison with the CTRL group, in which the lesion slightly increased due to acid challenge of the pH-cycling model. However, the toothpastes used showed statistically significant differences in terms of remineralization potentials. It is important to highlight that all toothpastes contain fluoride concentration of 1450 ppm, with differences in the ingredients, which display different solubility, influencing the bioavailability of fluoride in saliva and, as a consequence, the remineralization potential.

SF group, which reported from mechanical, chemical, morphological, and elemental points of view the greatest remineralization potential, was treated with a sodium fluoride-based toothpaste, that has been currently shown to be helpful in remineralization [39]. Indeed, when saliva comes in contact with the toothpaste during brushing, calcium, phosphate, and fluoride are deposited on the tooth surface, forming calcium fluoride (CaF_2_) precipitates. At low pH, free fluoride can be released from the CaF_2_ deposit and subsequently reduce demineralization in the local microenvironment. On the other hand, when the pH increases, the presence of free fluoride serves to drive mineral formation, thus promoting remineralization [39–41]. SF contains also enzymes, such as glucose oxidase, amyloglucosidase, and lactoperoxidase, which stimulate natural salivary defenses [42]. Indeed, a previous study reported that the enzyme cascade results in the production of hypothiocyanite and hydrogen peroxide. This could lead to a growth in oxygen levels and a reduction of the increase of anaerobic bacteria, leading to a shift in the bacterial community towards an overall healthier oral microbiome [43]. Since penetration of fluoride into enamel is limited and decreases exponentially with enamel depth, it might be possible that the incorporation of enzymes in the toothpaste, which reduce the pH in the oral cavity producing hydrogen peroxide, helps extend the depth of fluoride penetration, and therefore allows lesion remineralization [44]. Indeed, in terms of crystallinity, which represents the degree of order within the HA crystals in the enamel, according to our findings, and Ca/*P* values, which represent an index of remineralization, SF showed a similar trend compared to HAF, with a significatively increase in the post values respect to the pre ones. Moreover, both groups were found to be improving the HV, with the SF post-treatment values leaning more towards healthy enamel values, with respect to HAF and SMF [18]. However, as evidenced by SEM microphotographs, SF evidenced a higher ability in remineralizing the demineralized surface compared to both HAF and SMF, with a complete restoration of the lost interprismatic areas. Finally, from the µ-CT analysis, the lesion in SF appeared, after treatment, not only smaller in size, but also with an increased mean value of MD, obtained from a fitting curve applied to gray levels measured with the X-ray tomograph.

The HAF group was subjected to treatment with a toothpaste containing HA, which is recognized as one of the most biocompatible and bioactive materials. This is due to its resemblance to the apatite crystal of tooth enamel in morphology, crystal structure, and crystallinity [45]. The findings of this study were consistent with other in vitro research, which also demonstrated remineralization after using toothpastes containing nano-HA [46–48]. However, in our study, the HAF group reported a higher remineralization potential with respect to SMF, but a lower one in comparison with SF. This result could be attributed to the solubility properties of nanoHA; indeed, a previous study on the remineralization effect of nanoHA toothpaste on artificial caries reported that the solubility properties of nanoHA play a significant role in remineralization when the demineralized specimens were subjected to the treatment solutions continuously for several days [45]. Thus, since the toothpaste was applied for a short period in this study, probably not enough Ca^2+^ and PO_4_^3−^ were available to increase the stability of HA in the enamel and to give greater remineralization potential to the WSLs.

Finally, the SMF group was treated with sodium monofluorophosphate toothpaste, associated to arginine. A previous study reported that arginine has been introduced in association with sodium monofluorophosphate, in order to optimized for remineralization [49]. In line with the concept of organic-to-inorganic interactions observed in tissue mineralization, arginine, with its positively charged properties, acts as an organic nucleation center for mineralization [50]. In accordance with previous studies, this product demonstrated its remineralizing effect; however, it resulted in lower respect to sodium fluoride and HA-based toothpastes.

Based on our results, the proposed null hypothesis, i.e., that the tested materials did not show differences in the remineralizing potential of WSLs, was partially refused. In fact, all the toothpastes showed a remineralization capability of WSLs after 7 days of treatment, but the best performances were displayed by the SF and HAF groups.

The limitations of this in vitro study are related to the experimental design, as it is difficult to simulate the in vivo conditions of the oral cavity [38]. Indeed, this study has a restricted ability to replicate the complexity of plaque, saliva pellicle, and bacterial biofilms found in the oral cavity, which could have a role during the remineralization and demineralization processes. Then, the use of samples from different donors may have been influenced by varying environmental exposures to diet, resulting in diverse responses under acidic-challenged conditions. Moreover, the experimental periods for remineralization were shorter than the expected period in real-life, in vivo conditions [38], therefore future studies will investigate longer time-periods.

From a clinical point of view, these results could give the practitioners useful clinical indications regarding the management of WSLs. Through the obtained findings, all the tested agents, but above all sodium fluoride toothpaste associated with enzymes, are potentially effective as remineralizing agents and could be efficient in preventing dental caries.

These findings also suggest their application in other dental tissue diseases concerning the demineralization of the enamel, such as molar incisor hypomineralization, which could be a potential area for further investigations.

## Conclusion

Remineralization treatment is a modern non-invasive approach for early carious lesions. This is critical for dental clinicians as it enables a shift in their therapeutic strategy towards a new paradigm focused on prevention and minimally invasive interventions. The present study demonstrated that toothpastes based on HA partially replaced with fluoride, sodium monofluorophosphate with arginine, and sodium fluoride toothpaste associated with enzymes have the potential to remineralize the WSLs. In particular, sodium fluoride toothpaste associated with enzymes showed the best results in terms of remineralizing potential. Since the toothpaste is used daily by the patient, the tested products represent an effective everyday tool for treating the WSLs.

## Data Availability

All data underlying the results are available as part of the article and no additional source data is applicable. The data presented in this study are available on request from the corresponding author.
